# How many days are needed? Measurement reliability of wearable device data to assess physical activity

**DOI:** 10.1371/journal.pone.0282162

**Published:** 2023-02-24

**Authors:** Patrick Hilden, Joseph E. Schwartz, Christian Pascual, Keith M. Diaz, Jeff Goldsmith

**Affiliations:** 1 Department of Biostatistics, Mailman School of Public Health, Columbia University, New York, New York, United States of America; 2 Center for Behavioral Cardiovascular Health, Department of Medicine, Columbia University Irving Medical Center, New York, NY, United States of America; 3 Department of Psychiatry and Behavioral Sciences, Renaissance School of Medicine, Stony Brook University, Stony Brook, NY, United States of America; 4 Department of Biostatistics, Herbert Wertheim School of Public Health, University of California, San Diego, San Diego, CA, United States of America; Mugla Sitki Kocman University: Mugla Sitki Kocman Universitesi, TURKEY

## Abstract

**Introduction/Purpose:**

Physical activity studies often utilize wearable devices to measure participants’ habitual activity levels by averaging values across several valid observation days. These studies face competing demands–available resources and the burden to study participants must be balanced with the goal to obtain reliable measurements of a person’s longer-term average. Information about the number of valid observation days required to reliably measure targeted metrics of habitual activity is required to inform study design.

**Methods:**

To date, the number of days required to achieve a desired level of aggregate long-term reliability (typically 0.80) has often been estimated by applying the Spearman-Brown Prophecy formula to short-term test-retest reliability data from studies with single, relatively brief observation windows. Our work, in contrast, utilizes a resampling-based approach to quantify the long-term test-retest reliability of aggregate measures of activity in a cohort of 79 participants who were asked to wear a FitBit Flex every day for approximately one year.

**Results:**

The conventional approach can produce reliability estimates that substantially overestimate the actual test-retest reliability. Six or more valid days of observation for each participant appear necessary to obtain 0.80 reliability for the average amount of time spent in light physical activity; 8 and 10 valid days are needed for sedentary time and moderate/vigorous activity respectively.

**Conclusion:**

Protocols that result in 7–10 valid observation days for each participant may be needed to obtain reliable measurements of key physical activity metrics.

## Introduction

Studies that use wearable devices often produce daily summary metrics like time spent in sedentary, light (LPA), and moderate to vigorous physical activity (MVPA), in order to quantify daily physical activity for study participants. Recognizing that there is day-to-day variability in activity within participants, individual daily observations are usually aggregated by averaging across days to obtain better, more robust estimates of each person’s average daily physical activity. While it is clear that averaging over a greater number of days of observation will yield a more stable estimate of an individual’s daily average, it is not well known how many days of observation are “enough”–that is, what number of days will be sufficient to produce an aggregate measure that reflects actual habitual activity levels. Previous studies have framed this as a question of test-retest reliability, and used related methods to estimate the number of observation days needed to meet a pre-specified reliability threshold [1–5]. However, it is not obvious that one can generalize from consecutive day-to-day measurements to longer-term test-retest reliability of a person’s average physical activity, and no studies to our knowledge have used long-term follow-up data to determine the actual long-term test-retest reliability of aggregate measures provided by data collected over a pre-specified observation period.

Measurement reliability was originally introduced and is the subject of a rich literature in the field of psychometrics, and has since seen applications in a variety of other areas [6–10]. The framework used in the context of physical activity metrics assumes that each participant has an underlying true value, with measurements differing from this true value due to independent, identically distributed random deviations. This assumes, for example, that each participant has his or her own true habitual sedentary time, and each day’s measurement is a completely random deviation from that true time. Given multiple daily measurements on participants for an outcome of interest (e.g. total sedentary minutes for each participant and day over a week), reliability is defined as the ratio of between-person variability to total variability, ranging from 0 to 1 [11]. Reliability is high when the magnitude of the random deviations is small relative to the differences between participants. A generally accepted standard for good reliability is 0.80, and measurements with lower reliability may contain more random variability than desired for subsequent analysis.

Somewhat counter-intuitively, reliability describes a feature of a single measurement–what information a single day’s sedentary time would contain if no other days were available–even though it requires multiple observations to estimate. In the context of physical activity, it is further desirable to consider the reliability of an observation obtained through aggregating/averaging multiple repeat measurements. Given an estimated reliability for a single observation, the Spearman-Brown prophecy formula is designed to determine the number of observations per person which, if averaged, would result in a pre-specified level of reliability for this average, which we refer to as “aggregate reliability”. Based on application of the prophecy formula to data from a number of previous studies, current recommended practice for the determination of habitual physical activity via accelerometry is to aggregate measurements from 3–5 days among adults, and 4–9 days among children [12, 13]. Accordingly, 7-day accelerometer protocols have become conventional in the field to meet these goals while allowing for some non-wear days [14–18].

There are a number of limitations to providing an estimated number of days needed to achieve an aggregate reliability of 0.80 based on the prophecy formula. First, the number of days derived from the prophecy formula is an estimated value, but variability in this estimate (e.g., a confidence interval) has been underreported. Second, the underlying statistical assumptions surrounding the estimation of reliability, including the independence of measurement days within participants and the homogeneity of variances of deviations both within and across participants, may be unmet in practice. Finally, just as multiple observations are needed to understand the reliability of a single observation, it is necessary to obtain multiple independent aggregate measures to empirically demonstrate their reliability; without that follow-up, it is unclear if multi-day averages achieve their prophesied reliability. If the actual reliability is lower than the prophesied reliability, estimates of the relationship of physical activity with other variables are likely to be attenuated and studies based on a presumed reliability of 0.80 may be underpowered.

The purpose of this work is to better understand the variability associated with estimating reliability in practice, the corresponding results of applying the prophecy formula, and the actual reliability of aggregate measures. We use data from a long-term follow-up study that collected device-measured physical activity data for up to 365 consecutive days, and design an empirical study of single-measure and aggregate reliability. We consider several observation windows when examining aggregate reliability, which may inform best practice recommendations regarding accelerometer protocol lengths.

## Materials and methods

### Ethics

The study protocol was approved by the Institutional Review Board of the Columbia University Irving Medical Center. Data on student participants was collected after informed written consent; additional consent for the secondary analysis of deidentified data in this study was waived.

### Study design

The motivating data for our evaluation come from a 12-month randomized controlled trial, conducted at the Center for Behavioral Cardiovascular Health at the Columbia University Irving Medical Center, which sought to better understand the bi-directional relationship between physical activity and stress. Participants were recruited using fliers posted throughout the Columbia University Irving Medical Center; were 18 years or older; reported only intermittent engagement in exercise; did not have comorbidities that would prevent or compromise engagement in physical activity; did not have work demands requiring vigorous activity; and would be available during the following continuous 12 months [19].

The 79 participants in this study were asked to wear an activity monitor (Fitbit Flex) on their wrist every day for approximately one year. After six months of data collection, participants were randomized to receive either general information about their exercise and reported stress or a personalized ‘stress-exercise fingerprint’ detailing 2–4 personal predictors of engaging in exercise identified from the initial 6 months of observational data [19, 20]. As discussed below, the majority of data used in the primary analysis (and all data used in the sensitivity analysis) were collected prior to the intervention, and for that reason should be considered observational in nature.

### Device-based measurement of physical activity

Physical activity was measured using a wrist-based model of the Fitbit (Fitbit Flex; Fitbit, Inc., San Francisco, CA). The Fitbit Flex is a microelectromechanical triaxial accelerometer that has been demonstrated to be valid and reliable for measuring physical activity and sedentary behavior in adults [21–23]. Data from the device automatically uploads to the Fitbit website whenever the device is within 15 feet of a smartphone with the accompanying Fitbit application installed or a base station, which for this study was plugged into the participant’s own computer. Participants were instructed to sync and charge their device every 5–7 days to ensure no loss of activity data. The Fitbit Flex was selected because it is simple to use and more convenient than research-grade accelerometers which require the participant to return to the study office for syncing and battery charging/replacement.

The minute-by-minute activity data were extracted from the manufacturer’s website using the Fitabase software. Our analysis focuses on the typical waking hours of 8:00am to 10:00pm. Any two-hour period where no steps were observed was considered to be nonwear [24], and we required a minimum of 10 hours of wear time over the 14-hour observation period for a given day to be considered valid and therefore included in our analysis. For each participant, we excluded the first 14 days of observation as a run-in period to allow for the possibility that an individual’s activity may differ during this time due to device reactivity [25]. After discarding the run-in period, we selected the next 60 valid days of observation for each participant; this provides a period that is long enough to obtain multiple non-overlapping observation windows to study reliability, but short enough that habitual activity levels were unlikely to be affected by substantial behavioral changes.

For each valid day we used the device step counts for each 1-minute epoch (e.g. step cadence) to determine the total number of sedentary (steps = 0 for a given 1-minute epoch), light (0 < steps < 100 for a given 1-minute epoch), and moderate/vigorous (steps ≥ 100 for a given 1-minute epoch) minutes of activity. We used step counts in our definition of intensity, rather than device-produced intensities, due to some lack of information regarding the device’s intensity algorithm, the implausibility of some of the produced values, and its corresponding validity for distinguishing physical activity intensity (particularly for sedentary and LPA time). Our choice of thresholds uses an established approach to defining intensity based on per minute step counts to infer step cadence [26, 27]. Adjustment for day-to-day variability in total wear time between 8:00am and 10:00pm was done via a linear model weartime correction, with a single model used for the full cohort [28].

### Statistical analyses

#### Review of prior approaches to assessing reliability

Reliability quantifies the degree of similarity of observations within a person, with the implicit assumption that measurements are taken in unchanging conditions. It is assumed that individual measurements are the combination of a true person-level value and random deviations from that. The model assumed to generate an observed measurement *X* is

X=T+e (1)

where *T* is the underlying true value intended to be measured and *e* is a random deviation from that value. It is assumed that *T* has mean *μ* and between-person variance $\sigma_{T}^{2}$ while *e* is a mean zero residual with constant variance $\sigma_{e}^{2}$ that is the same within and between participants; the residual has mean zero under the assumption that the measurements are unbiased for the true value. Further, it is assumed that *T* and *e* are independent and that the *e* are uncorrelated with each other both within and between participants (r(*e*_*tj*_, *e*_*tk*_) = 0 for all j ≠ k). The reliability of a single measurement *R*_1_, then, is defined as the percentage of overall variability that is due to true person-to-person differences:

$$R_{1}=\frac{var(T)}{var(X)}=\frac{\sigma_{T}^{2}}{\sigma_{T}^{2}+\sigma_{e}^{2}}.$$ (2)

This framework emphasizes that, conceptually, reliability depends on partitioning individual measurements into true scores and noise and similarly partitioning the total variance into the variance of the true scores (between-person variance) and the variance of the random deviations (within-person variance).

In practice, estimating reliability depends on data with multiple measurements per person, giving rise to observations

$$X_{ij}=T_{i}+\epsilon_{ij}$$ (3)

with participants *i* = 1,…,*n* and replicates *j* = 1,…,*J*. Given such data, measurement reliability *R*_1_ can be estimated using a mixed effects model with a random intercept for each person (in this setting, *R*_1_ is widely known as the intraclass correlation coefficient, or ICC). The mixed model produces estimates ${\hat{\sigma }}_{T}^{2}$ and ${\hat{\sigma }}_{e}^{2}$, which in turn can be used to estimate the reliability ${\hat{R}}_{1}$.

It is well known that the average of a set of independent and identically distributed measurements of the same quantity provides a more accurate estimate of the underlying true value of interest. Given independent and identically distributed replicate measurements for each person, the aggregate reliability of the average of *J* measurements (${\bar{X}}_{i}=\frac{1}{J}\sum_{j=1}^{J}X_{ij}$) is given by

$$R_{\bar{J}}=\frac{var(T_{i})}{var({\bar{X}}_{i})}=\frac{\sigma_{T}^{2}}{\sigma_{T}^{2}+\sigma_{e}^{2}/J}.$$ (4)

As the number of replicates *J* increases, there is a corresponding increase in aggregate reliability $R_{\bar{J}}$. This relationship can be reexpressed as a function of the reliability of a single measurement via

$$R_{\bar{J}}=\frac{JR_{1}}{1+(J-1)R_{1}}.$$ (5)

The preceding is known as the Spearman-Brown prophecy formula, which relates the reliability of a single measurement, *R*_1_, and the reliability of the average of *J* measurements, $R_{\bar{J}}$. The prophecy formula has been utilized in physical activity studies to obtain an estimate $\hat{J}$ of the number of days *J* that are necessary to produce a desired level of aggregate reliability, most typically $R_{\bar{J}}=0.80$, for activity metrics like average daily sedentary, LPA, and MVPA time.

This validity of this application of the prophecy formula to extrapolate from short-term consistency to long-term aggregate reliability rests on model assumptions described above that may not hold in practice. In short, it is assumed the observations *X*_*ij*_ are independent, both within and across participants; that the residual variance $\sigma_{e}^{2}$ is constant across repeated observations and the same for all participants; and that a person’s true underlying value is constant and reflects habitual activity. However, it is unlikely that activity across consecutive days are truly independent within a person. Further, the residual variability may differ across participants and also within a person over time. Finally, it is unclear whether it is reasonable to assume a habitual level of activity, or over what timeframe this might be valid. For these reasons, the direct use of Eq 1 and the prophecy formula may be inappropriate for physical activity measurements. When applied to activity data, violations of these assumptions can produce inaccurate estimates of reliability *R*_1_ and, by extension, $R_{\bar{J}}$.

Recall that to accurately assess the reliability *R*_1_ of a measurement *X*, it is necessary to obtain several independent observations across multiple participants. A similar argument applies to the reliability $R_{\bar{J}}$ of an aggregate measure $\bar{X}$: although the Spearman-Brown formula is valid when all assumptions are met, it is prudent to estimate the reliability of the aggregate measure directly by obtaining and analyzing independent replicates of the aggregate measure. A marked difference between the value obtained this way and the one derived from the prophecy formula would suggest that the observed data are not consistent with one or more of the assumptions underlying the prophecy formula. To our knowledge, however, a direct examination of $R_{\bar{J}}$ for measures of sedentary, light and moderate/vigorous physical activity has not been conducted.

#### Resampling strategy to evaluate reliability

We addressed two specific aspects of measurement reliability for physical activity data. First, we evaluated the estimation of reliability and the use of the prophecy formula when replicate measurements are observed over a single period. Second, we assessed the reliability of the average of several observations by taking such averages in two distinct observation windows. The first analysis was intended to provide more insight into how reliability has been estimated in existing work based on single observation periods, and the second analysis was intended to clarify the relationship between prophesied and actual reliability. Through addressing these specific aspects, the present study also informs how many valid days of wear are needed to obtain reliable (>0.80) assessments of the different physical activity metrics via wearable devices.

**Fig 1:**
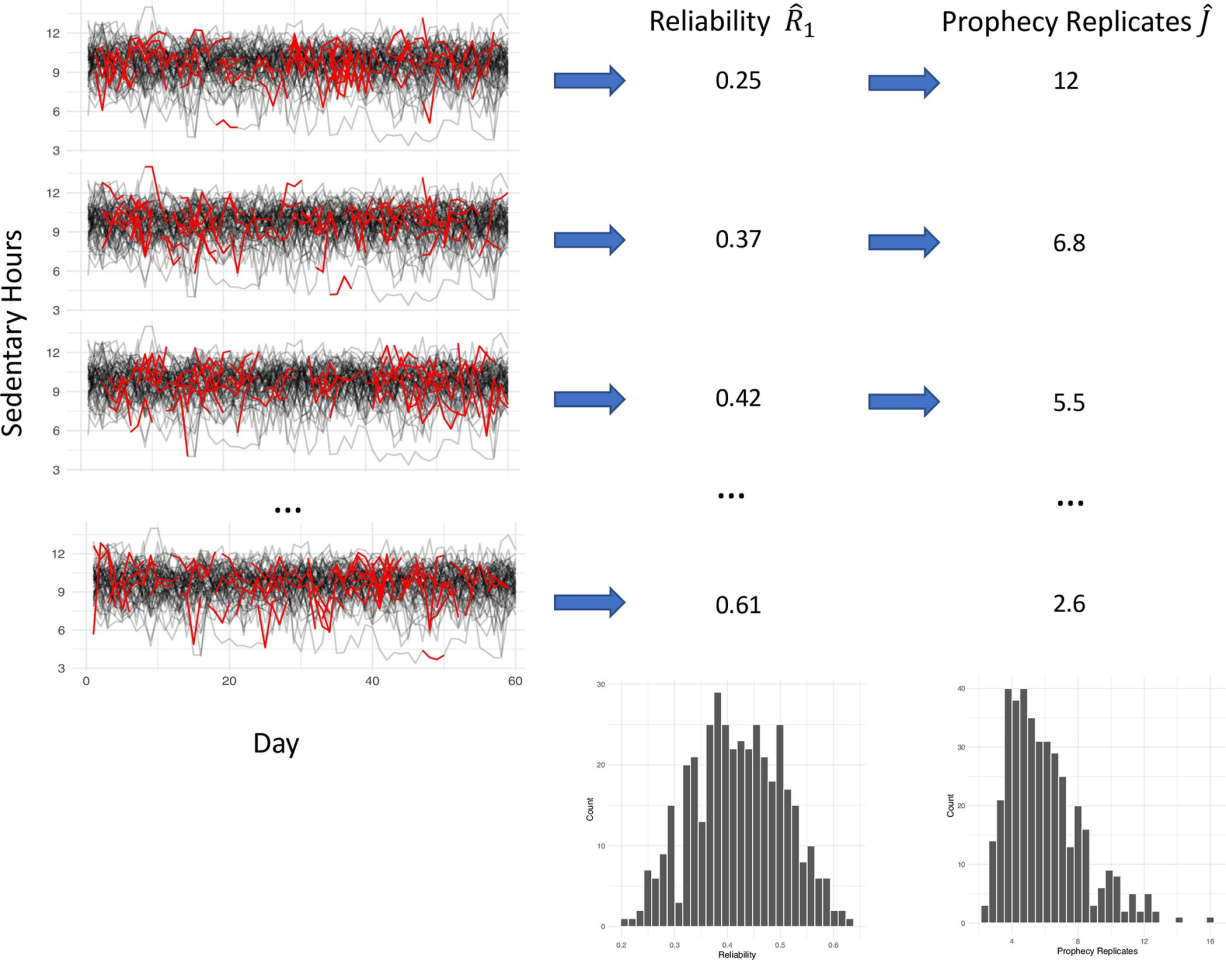
Framework for creating sampled datasets. Each row represents a single sampled dataset. The left column contains all of the observed activity trajectories in the motivating data, with the red segments representing the selection of days *J* which were selected for a given sample. The second column shows the estimated reliability ${\hat{R}}_{1}$ based on the previous selection of days. The final column shows the subsequent prophecy estimate of the number of replicates $\hat{J}$ required to achieve a reliability of 0.8. The histograms at the bottom of the second and third columns represent the distributions of ${\hat{R}}_{1}$ and $\hat{J}$ across all 200 samples generated in this way.

For the first set of analyses, we evaluated the variability of estimates of both *R*_1_ and the number of days needed to obtain an aggregate reliability of 0.80 according to the Spearman-Brown prophecy formula. This sampling variability was quantified by randomly selecting an observation window for each person of *J* days and calculating the single-measurement reliability ${\hat{R}}_{1}$ associated with sedentary, LPA, and MVPA time. Given the estimated reliability ${\hat{R}}_{1}$, the Spearman-Brown prophecy formula was then applied to estimate the number of days ${\hat{J}}_{0.80}$ which would need to be averaged to achieve an aggregate reliability $R_{\bar{J}}=0.80$. This process mimics the estimation of reliability and the application of the prophecy formula used in previous studies. These steps were repeated 200 times, each time selecting a new observation window for each participant, for *J* = 2,3,…,10 valid days in order to obtain a sampling distribution of ${\hat{R}}_{1}$ and ${\hat{J}}_{0.80}$. Fig 1 contains a conceptual diagram of this process when performed for sedentary time.

Next, we determined the actual reliability for the averages of *J* valid days. We investigated this by first selecting two distinct periods of *J* days per person and averaging sedentary, LPA, and MVPA time within both periods. We additionally required that the two periods be at least 7 days apart; this reduces potential within-person correlation between the two distinct periods, although days within periods may still be correlated. In this way, we obtained two independent average measures of each activity metric, with averages based on observation windows of *J* days. Using these averages we then estimated ${\hat{R}}_{\bar{J}}$ directly as the intraclass correlation of the two averages. This process was repeated 200 times each for *J* = 2,3,…,10 days.

## Results

Of the seventy-nine participants recruited for the original study, ten did not have at least 60 valid days of observation and were excluded from our analysis. Table 1 contains demographic characteristics of the sample used for primary analysis.

**Table 1:** Demographics in analytical sample.

Characteristics	Mean (SD) or N (%)
Average age, y	32.3 (9.8)
BMI (kg/m^2^)	26.8 (5.3)
Gender	
Men	29 (42.0%)
Women	40 (58.0%)
Race	
Asian	15 (21.7%)
Black/African American	10 (14.5%)
Native Hawaiian/Pacific Islander	1 (1.4%)
White	28 (40.6%)
2 or more	3 (4.3%)
Unknown/Declined (mostly Hispanic)	12 (17.4%)

**Fig 2:**
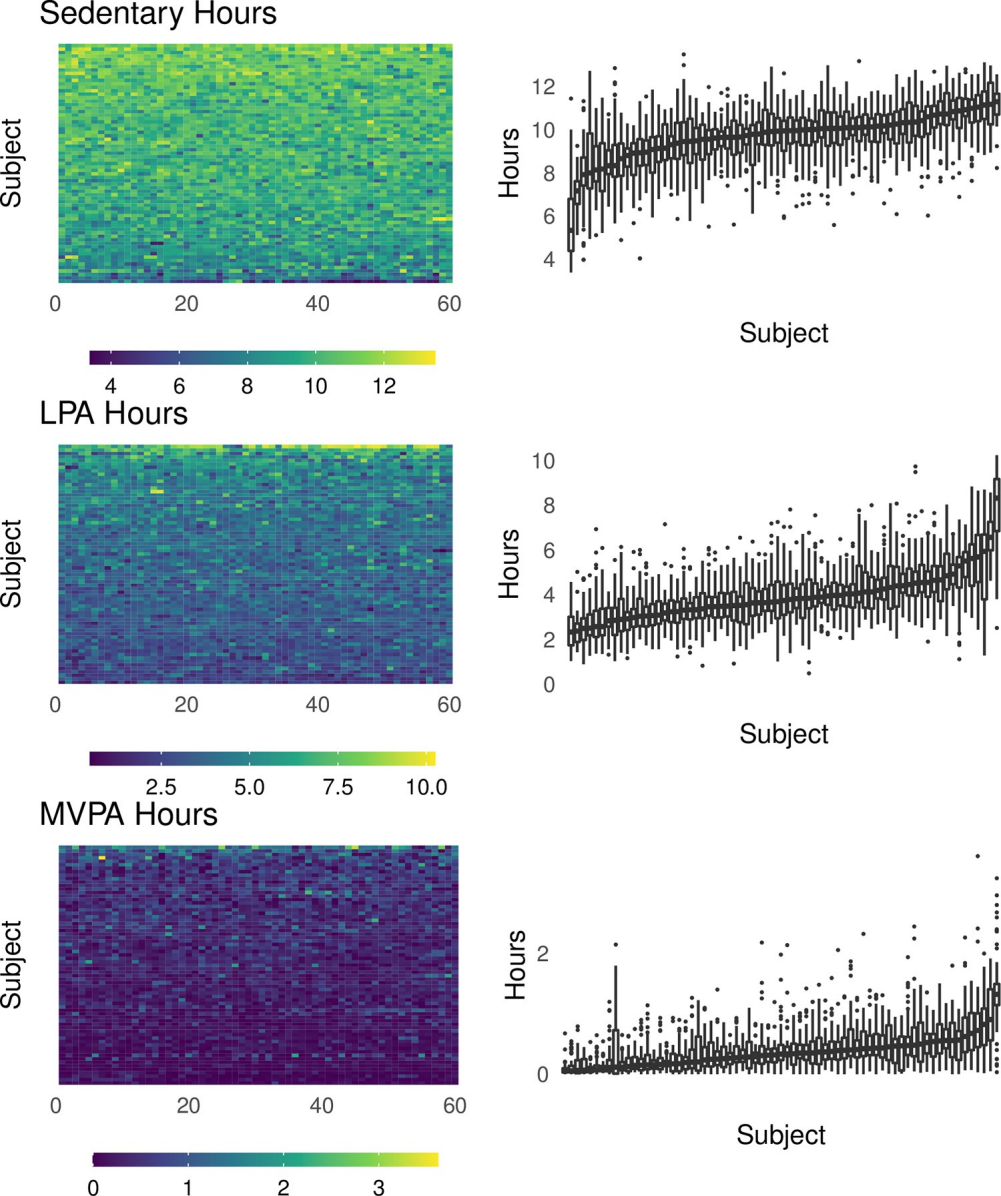
Activity metric distributions in the motivating data set. The left column of panels shows the heatmaps for each metric and participant over the 60 days of observation included in the present analysis. The right column shows boxplots of sedentary, light, and MVPA time for each participant over the course of the study, sorted based on the median value for each metric separately. Individual boxes show bars at the median value, hinges at the 25% and 75% quantile, and whiskers extending to observed values within (hinge ± 1.5 * IQR). Data points outside the range of whiskers are shown.

The mean sedentary and LPA hours per day were 9.7 (SD = 1.4) and 3.9 (SD = 1.4), respectively, and distributions of these values were roughly symmetric within individuals. The median MVPA hours per day was 0.32 (IQR = [0.10, 0.53]). The median number of days required to achieve 60 valid days of observation was 81 (range = [60, 330]; IQR = [72, 98]). The resulting dataset is illustrated in the top row of panels in Fig 2, which shows each individual’s activity metrics over the 60 days of observation. This Figure highlights that there is both day-to-day variation within a participant and person-to-person variation, both of which affect measurement reliability. Visual inspection of Fig 2 also suggests that any systematic temporal changes within participants, if they exist, are small relative to the within- and between-person variation.

### Estimate variability

**Fig 3:**
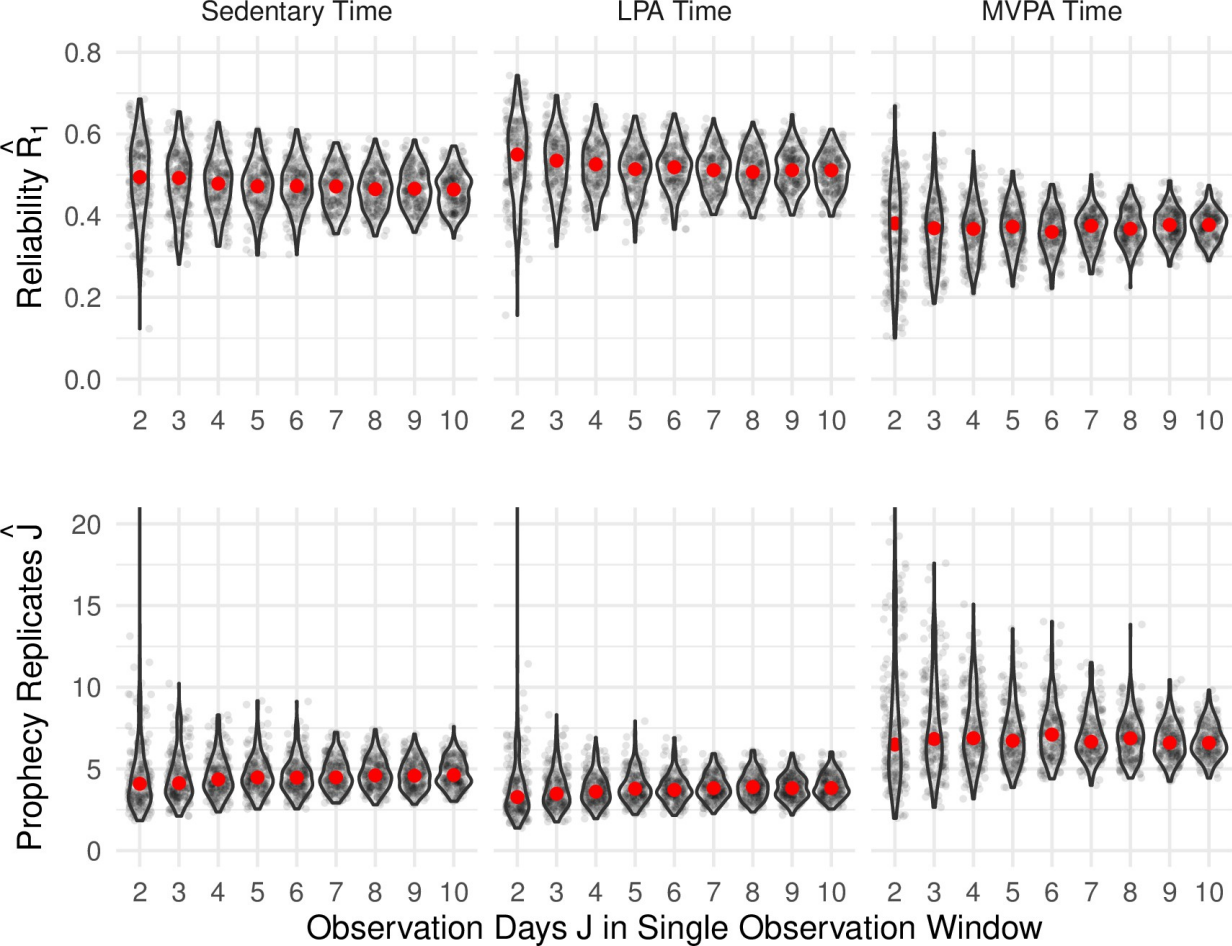
Estimated reliability and number of days. The top row shows the empirical distribution of estimated reliability ${\hat{R}}_{1}$ for sedentary, LPA, and MVPA time. Each panel shows the distribution of reliability estimates ${\hat{R}}_{1}$ based on observation periods of between 2 and 10 days with individual points showing the results for a single generated dataset. The bottom row shows the corresponding empirical distribution of the number of replicates ${\hat{J}}_{0.80}$ based on the prophecy formula required to achieve *R*_*J*_ = 0.80 for each activity metric and observation window. Solid red circles indicate the median across 200 sampled datasets.

The results in Fig 3 emphasize that there is considerable sampling variability in the estimates of *R*_1_ and *J*, especially for lower numbers of observation days. As expected, with a larger number of days we see decreased variability in the estimation of both values. Across the activity metrics, reliability is highest for LPA time; correspondingly, the estimated number of required replicates $\hat{J}$ indicated by the prophecy formula is smallest for LPA time. For example, the top center panel of Fig 3 shows that given 7 days of observation per person, the median reliability estimate for LPA time was 0.51 (IQR = [0.47, 0.54]), with a minimum observed reliability of 0.40 and a maximum of 0.64. The bottom center panel then shows that given 7 days of observation per person, the median number of replicates, *J*, required to achieve *R*_*J*_ = 0.80 based on the prophecy formula was 3.8 (IQR = [3.4, 4.5]) with a minimum of 2.3 and maximum of 5.9. In contrast, the reliability ${\hat{R}}_{1}$ for MVPA obtained from a single sampled dataset is often lower than 0.4, and values for ${\hat{J}}_{0.80}$ are higher than 6. The values for *J* obtained across sampled datasets are more variable for MVPA than for LPA or sedentary time, which may derive from the heterogeneity within and across participants seen in Fig 1.

### Direct estimation of aggregate reliability

**Fig 4:**
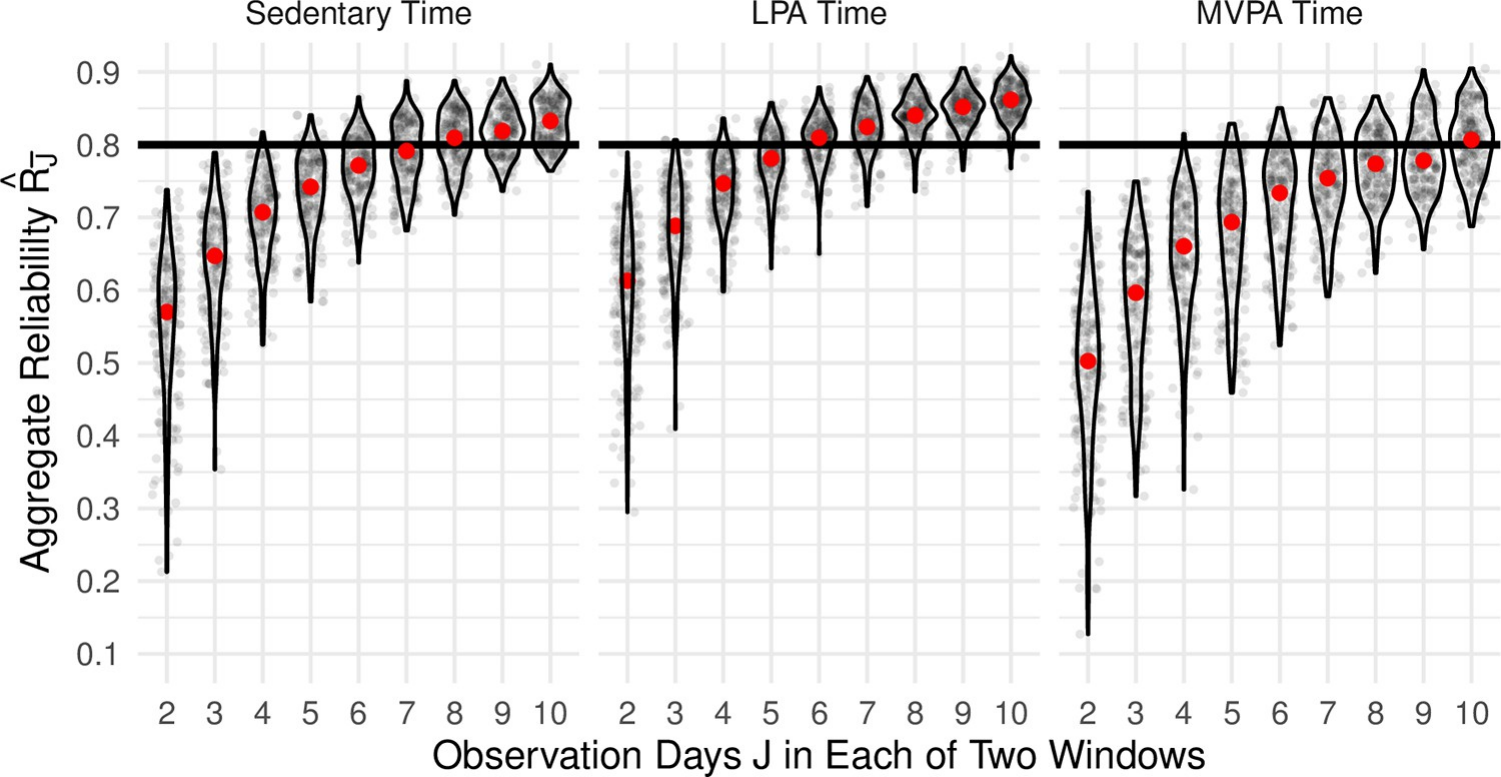
Direct estimation of aggregate reliability. Results of the investigation into aggregate reliability of the average daily activity for each activity metric, estimated as the test-retest reliability (intraclass correlation) of the averages from two separate periods of between 2 and 10 days selected for each participant. The solid red circle indicates the median across 200 sampled datasets.

Fig 4 provides the results of our second analysis, in which two separate periods of *J* days were averaged for each participant to directly estimate the test-retest reliability of ${\hat{R}}_{\bar{J}}$.

As expected, the aggregate reliability ${\hat{R}}_{\bar{J}}$ increased as the number of observation days *J* increased and the variability in ${\hat{R}}_{\bar{J}}$ decreased. Aggregate reliability was highest for LPA time, with the center panel of Fig 4 showing that for 7 days of observation the median ${\hat{R}}_{\bar{7}}$ was 0.83 (IQR = [0.80, 0.85]), with a minimum of 0.72 and a maximum of 0.89, with 77% of ${\hat{R}}_{\bar{J}}\geq 0.80$. Aggregate reliability for Sedentary time was somewhat lower: 7 days of observation resulted in a median ${\hat{R}}_{\bar{7}}$ of 0.79 (IQR = [0.76, 0.83]). Aggregate reliability was lowest for MVPA time, with a median ${\hat{R}}_{\bar{7}}$ of 0.75 (IQR = [0.71, 0.79]) for 7 days of observation, and only 22.5% of ${\hat{R}}_{\bar{J}}\geq 0.80$.

For sedentary time, 8 days were required in order to achieve a median reliability ≥0.80 across our sampled datasets. Six and 10 days were needed in order to achieve a median reliability ≥0.80 for LPA and MVPA time, respectively.

## Sensitivity analysis

The number of days required to achieve 60 valid days of observation was high for some participants, and substantial behavioral changes affecting reliability may be more likely for these people. We therefore conducted a sensitivity analysis that included only the 53 participants who achieved 60 valid observation days in 100 days or fewer; 16 participants included in the prior analyses were excluded. The results of these sensitivity analyses are consistent with our primary findings; versions of Figs 2–4 using only this subset are shown in Supporting Information.

## Discussion

Studies that use wearable devices to assess physical activity face a number of constraints that limit the number of observation days that can be gathered for each participant. In order to design these studies, researchers seek to collect sufficient data to ensure that resulting aggregate measurements provide reliable estimates of participants’ habitual activity. Current recommendations were established by applying the Spearman-Brown prophecy formula to estimated reliability values obtained from a single observation window. Our work had two primary goals, which were made possible through a long-term study of physical activity. First, we sought to use the long-term nature of our data to better understand the properties of reliability and the prophecy formula as they have been used in the past. Second, we sought to assess aggregate reliability directly, through the use of multiple independent aggregate measurements obtained from each participant.

The results of our first investigation wherein we used the conventional approach to estimate reliability values via the prophecy formula are broadly consistent with previous recommendations. Application of the prophecy formula to the present data suggests that 3–5 days of observation would be adequate to provide an aggregate reliability of 0.80 for sedentary and LPA time, and that 6–8 days of observation would be reasonable for MVPA time. Our work gives additional insight into the uncertainty in estimating reliability and the number of days necessary to obtain 0.80 aggregated reliability in a future study. Our results also highlight that these conclusions are dependent on the activity metric of interest.

The results of our second investigation, however, suggest that the current approach to assessing reliability is flawed. In particular, the actual test-retest reliability of an aggregate measure based on 3–5 valid days is markedly lower than 0.80. Indeed, 6 observation days were needed to achieve a median reliability ≥0.80 for LPA time, and 10 observation days were needed for MVPA time. These results indicate that a 7-day observation protocol may be insufficient for sedentary and MVPA time, particularly when allowing for non-compliance or invalid observation days. Longer studies will be necessary to achieve an expected reliability ≥0.80 across physical activity metrics.

The contrast between results from our first and second approaches for assessing reliability may not be as surprising as they initially appear. Application of the Spearman-Brown prophecy formula is appropriate when the assumptions of the data generating model in Eq 1 are valid. Extending this framework to sequential measurements of activity over time is imperfect, likely due to a lack of independence of the repeated within-person measurements, non-constant variance across participants, and the possibility of an evolving “true value” over time. When assumptions are not met in practice, estimates of reliability based on single observation windows may be biased and overly optimistic. By constructing aggregate measures in two distinct time windows, we were able to obtain independent averages and assess aggregate reliability directly.

We note several important limitations of our analysis. We focus on a single long-term follow-up cohort consisting of young, healthy, mostly sedentary participants from a relatively homogeneous population. Reliability, by definition, is a population-specific measure; different results and guidelines should be expected for different or more heterogenous cohorts. Our analysis was based on a sample of 69 participants, and larger sample sizes would reduce some of the sampling variability we observed in Figs 3 and 4. That said, the median values of *R*_1_, *J*, and $R_{\bar{J}}$ and our conclusions about the duration of follow-up are unlikely to be substantially affected by the sample size. Participants generally complied with the study protocol, but our results could be confounded by factors that affect weartime and activity. Although there were few obvious changes in participants’ habitual activity, even small changes could impact measures of reliability. We suspect that issues of within-person correlation and non-constant variance across participants, together with fluctuations in habitual activity, are the main drivers behind the gap between prophesied and actual test-retest aggregate reliability. Analytic methods that account for these might improve estimation of *R*_1_ and *J* in data based on a single observation window and help close the observed gap. Lastly, we used step count data from a wrist-worn commercial device, and more work is needed to assess the reliability of other physical activity monitors and metrics, and for wearable devices that measure different biological processes.

This study focused on better understanding the framework that has been used for assessing the reliability of sedentary, LPA, and MVPA time. Our results suggest a mismatch between the assumptions underlying classic reliability theory and the Spearman-Brown prophecy formula and the real-world data generated in studies of physical activity. These may be addressed through improved analytic methods, but other critiques of the general approach will remain. Reliability *R*_1_ can be a difficult quantity to interpret in the context of physical activity, and aggregate reliability $R_{\bar{J}}$ even more so. Intuitively, a measurement with high (aggregate) reliability is likely to be similar across repeated observation: a reliable measurement of a participant’s average MVPA, for example, would be expected to vary relatively little from one observation period to another. More formally, measurements are reliable when most of the variation across participants is due to true systematic differences in their habitual physical activity. Because this definition of reliability depends on the ratio of between-person to total variability, the same measurement could be more or less reliable as the population in question changes. Finally, even reliable measurements will not reflect the underlying quantity in question if the measurement is not valid (i.e. if it does not measure the true physical activity or behavior of interest) or not accurate (i.e. if it consistently over- or under-estimates the phenomenon of interest). Tools and techniques that produce reliable, valid, and accurate measurements of physical activity are therefore necessary for the advancement of our understanding of the impacts of both sedentary behavior and physical activity on individuals’ health.

Past applications of the Spearman-Brown prophecy formula have found that the number of monitoring days that need to be averaged in order to achieve 0.80 reliability is between 3–5 and 6–8 for sedentary/LPA and MVPA time, respectively. However, our results suggest that the reliability of measurements averaged over monitoring periods of these durations will typically not result in an aggregate reliability of 0.80. In practice, a protocol that produces at least 8 valid days of observation is needed to assert that 0.80 reliability is likely to be met in terms of sedentary time, with protocols producing 6 and 10 valid observation days needed for LPA and MVPA time respectively. Significantly longer protocol lengths should be considered to confidently assert that a reliability of 0.80 has been (or will be) achieved.

## Supporting information

S1 Fig Activity metric distributions. The left column of panels shows the heatmaps for each metric and participant over the 60 days of observation for the sensitivity analysis. This can be compared to Fig 2 in the main manuscript.
(PDF)

S2 FigEstimated reliability and number of days in sensitivity analysis.The top row shows the empirical distribution of estimated reliability ${\hat{R}}_{1}$ for sedentary, LPA, and MVPA time. The bottom row shows the corresponding empirical distribution of the number of replicates ${\hat{J}}_{0.80}$ based on the prophecy formula required to achieve *R*_*J*_ = 0.80 for each activity metric and observation window. This can be compared to Fig 3 in the main manuscript.(PDF)

S3 Fig Direct estimation of aggregate reliability in sensitivity analysis. Results of the investigation into aggregate reliability of the average daily activity for each activity metric, estimated as the test-retest reliability (intraclass correlation) of the averages from two separate periods of between 2 and 10 days selected for each participant. This can be compared to Fig 4 in the main manuscript.
(PDF)
