# Supplementary figures and images for: How many days are needed? Measurement reliability of wearable device data to assess physical activity
Source: PLoS One. 2023 Feb 24;18(2):e0282162. doi: 10.1371/journal.pone.0282162 (PMC9956594; doi:10.1371/journal.pone.0282162)

Sedentary Hours

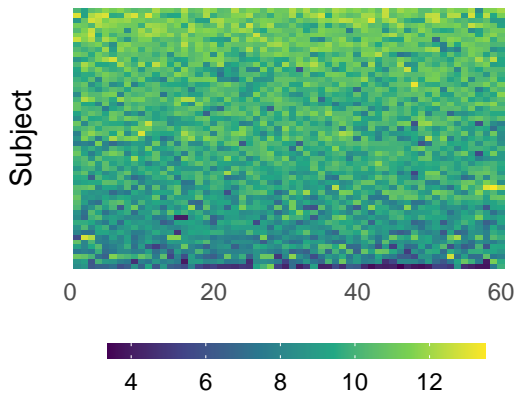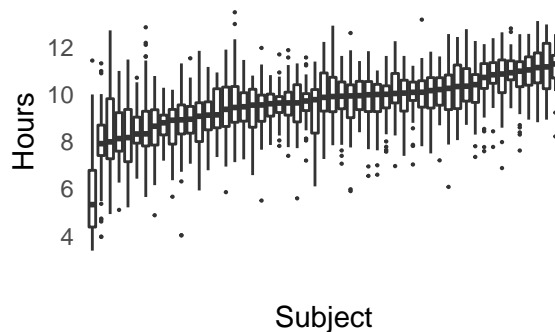

LPA Hours

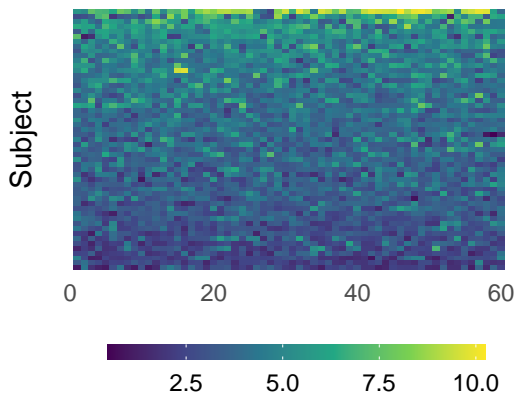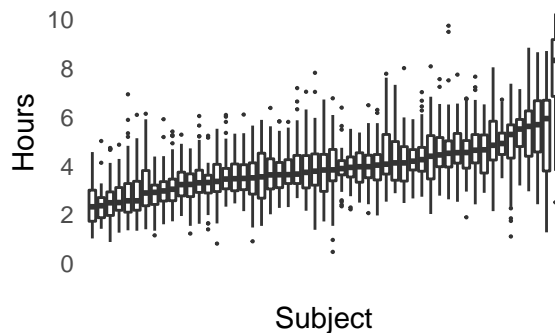

MVPA Hours

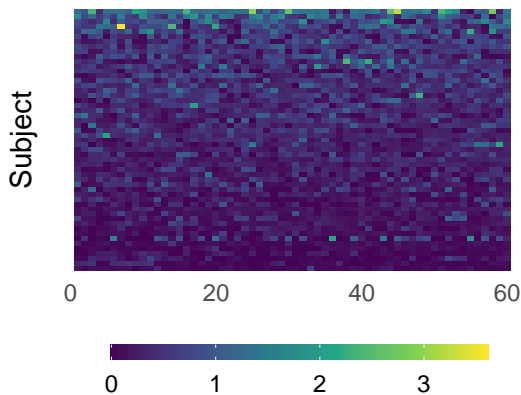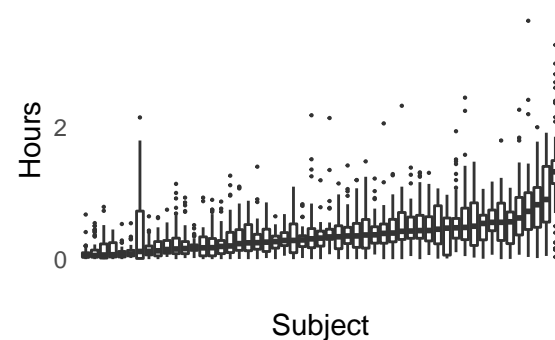

Supplement: S1 Fig — The left column of panels shows the heatmaps for each metric and participant over the 60 days of observation for the sensitivity analysis. This can be compared to Fig 2 in the main manuscript. (PDF) [file pone.0282162.s001.pdf]

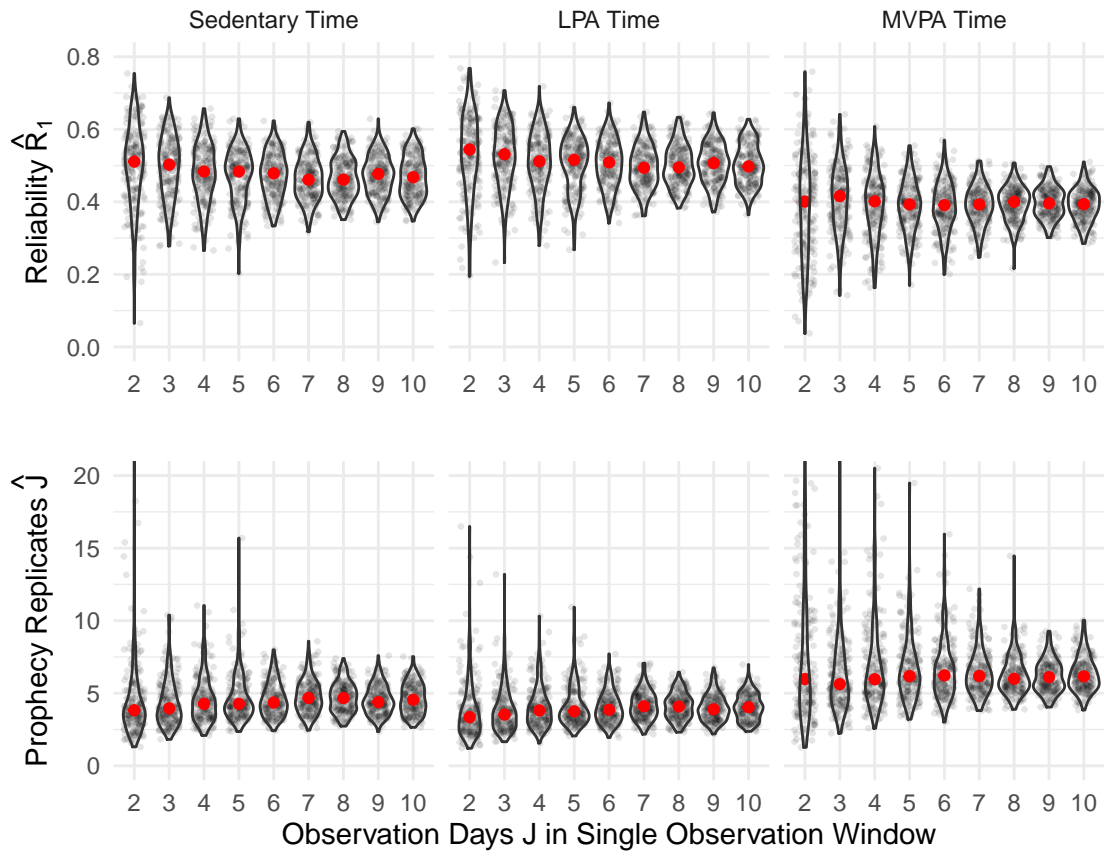

Supplement: S2 Fig — The top row shows the empirical distribution of estimated reliability R^1 for sedentary, LPA, and MVPA time. The bottom row shows the corresponding empirical distribution of the number of replicates J^0.80 based on the prophecy formula required to achieve RJ = 0.80 for each activity metric and observation window. This can be compared to Fig 3 in the main manuscript. (PDF) [file pone.0282162.s002.pdf]

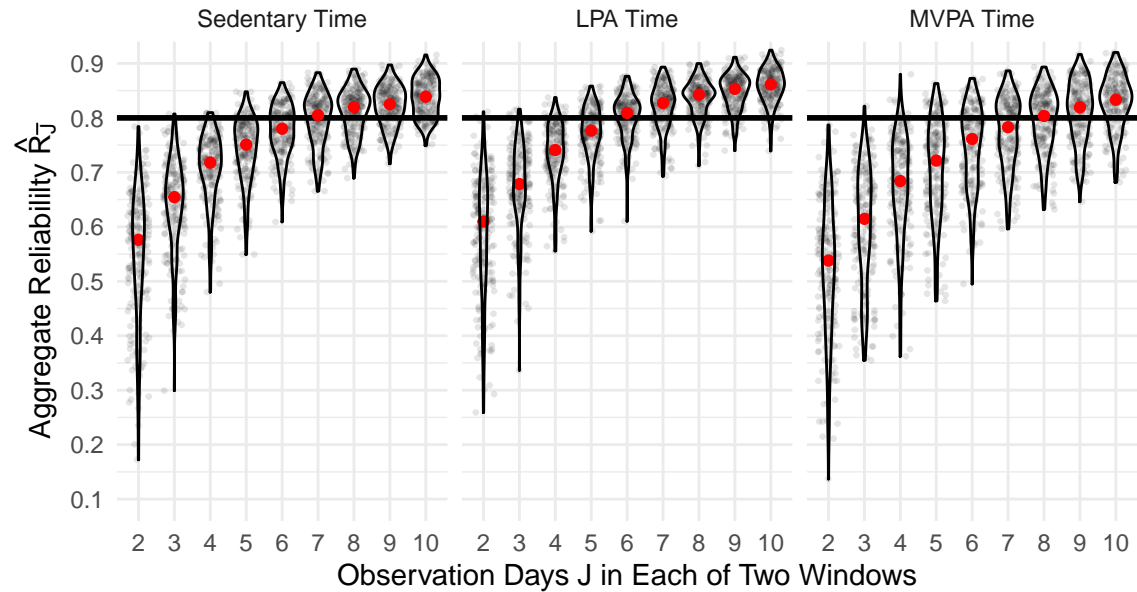

Supplement: S3 Fig — Results of the investigation into aggregate reliability of the average daily activity for each activity metric, estimated as the test-retest reliability (intraclass correlation) of the averages from two separate periods of between 2 and 10 days selected for each participant. This can be compared to Fig 4 in the main manuscript. (PDF) [file pone.0282162.s003.pdf]
